# Resin glycosides from Convolvulaceae plants

**DOI:** 10.1007/s11418-017-1114-5

**Published:** 2017-07-26

**Authors:** Masateru Ono

**Affiliations:** School of Agriculture, Tokai University, 9-1-1 Toroku, Higashi-ku, Kumamoto, 862-8652 Japan

**Keywords:** Resin glycoside, Jalapin, Convolvulin, Convolvulaceae, Glycosidic acid, Hydroxyl fatty acid

## Abstract

Resin glycosides are well known as purgative ingredients, which are characteristic of certain crude drugs such as Mexican Scammony Radix, Orizabae Tuber, and Jalapae Tuber, all of which originate from Convolvulaceae plants. Depending on their solubility in ether, these are roughly classified into two groups—jalapin (soluble) and convolvulin (insoluble). Almost all jalapins hitherto isolated and characterized had common intramolecular macrocyclic ester structures. These are composed of 1 mol of oligoglycoside of hydroxyl fatty acid (glycosidic acid) partially acylated by some organic acids at the sugar moiety, some examples of which are ester-type dimers. On the other hand, convolvulin is regarded as an oligomer of a variety of acylated glycosidic acids. This review describes the isolation and structural elucidation of resin glycosides from some Convolvulaceae plants, including *Ipomoea operculata*, *Pharbitis nil*, *Quamoclit pennata*, *Calystegia soldanella*, and *I. muricata*.

## Introduction

Resin glycosides are well known as purgative ingredients, which are characteristic of crude drugs such as Mexican Scammoniae Radix (the root of *Convolvulus scammonia* L.), Orizabae Tuber [the tuber of *Ipomoea orizabensis* (Pellet) Ledanois], and Jalapae Tuber (the tuber of *I. purga* Hayne). They are commonly found in plants belonging to the Convolvulaceae family [1]. Chemical investigations on these resin glycosides were initiated in the middle of the nineteenth century. The obtained resin glycosides were roughly classified into an ether-soluble group called jalapin or an ether-insoluble group denoted convolvulin [2]. Upon alkaline hydrolysis, both jalapins and convolvulins afforded organic acids such as isobutyric, 2-methylbutyric, (*E*)-2-methylbut-2-enoic (tiglic), 2-methyl-3-hydroxybutyric (nilic) acids and oligoglycosides of hydroxyl fatty acid (glycosidic acid). Furthermore, the glycosidic acid provided several kinds of monosaccharides (e.g., glucose, rhamnose, and fucose) and a hydroxyl fatty acid (e.g., 11-hydroxyhexadecanoic (jalapinolic), 11-hydroxytetradecanoic (convolvulinolic), and 3,11-dihydroxytetradecanoic (ipurolic) acids) on acidic hydrolysis. Considering this behavior toward acids and bases, Mannich and Schumann speculated that convolvulins from *I. purga* were oligomers of a glycosidic acid partially acylated by some organic acids at the sugar moiety [3]. However, until our research group isolated pure genuine jalapins, chemical studies had been confined only to the characterization of the component organic acids and glycosidic acids afforded by alkaline hydrolysis of a crude resin glycoside. In 1987, our research group succeeded, for the first time, in the isolation and structural elucidation of four jalapins from Orizabae Tuber [4]. Hitherto, almost all isolated and characterized jalapins have had intramolecular macrocyclic ester structures composed of 1 mol of oligoglycoside of hydroxyl fatty acids partially acylated by some organic acids at the sugar moiety, while some examples were ester-type dimers [5–13].

In this review, our studies on the isolation and structural elucidation of resin glycosides from some Convolvulaceae plants, including *I. operculata* (Gomes) Mart., *Pharbitis nil*, *Quamoclit pennata*, and *I. muricata*, are summarized.

### *I. operculata*

Rhizoma Jalapae Braziliensis (Brazilian Jalap), the root of *I. operculata* (Gomes) Mart., is known to be a substitute for Jalapae Tuber (Vera Cruz Jalap, *I. purga*), and its ethanol extract, Brazilian resin, is a potent laxative. Brazilian Jalap contains both jalapins and convolvulins as active ingredients.

Graf et al. reported that the alkaline hydrolysis of the jalapin fraction from Brazilian Jalap yielded acetic, tiglic, *n*-valeric, trimethylacetic, 2-methylbutyric, isovaleric, and propionic acids as component organic acids, while the convolvulin fraction (named rhamnoconvolvulin) afforded 4-oxodecanoic acid and 3,6:6,9-diepoxydecanoic (exogonic) acid, which are characteristic of Brazilian resin, along with those from the jalapin fraction [14, 15]. Wagner and Kazmaier isolated a major glycosidic acid named operculinic acid from rhamnoconvolvulin and characterized it as 3,12-dihydroxyhexadecanoic acid 12-*O*-α-d-glucopyranosyl-(1 → 4)-[*O*-α-l-rhamnopyranosyl-(1 → 6)]-*O*-α-d-glucopyranosyl-(1 → 3)-*O*-α-l-rhamnopyranosyl-(1 → 2)-[*O*-β-d-glucopyranosyl-(1 → 3)]-*O*-β-d-glucopyranoside [16].

#### Separation of crude jalapin and crude convolvulin fractions

The methanol (MeOH) extract of the root of *I. operculata* was partitioned between ether and H_2_O. The ether-soluble fraction was subjected to chromatography over MCI gel CHP 20P and Sephadex LH-20 columns to afford the crude jalapin fraction. The H_2_O-soluble fraction was partitioned between *n*-butanol and H_2_O. The *n*-butanol-soluble fraction was subjected to an MCI gel CHP 20P column to give the crude convolvulin fraction [17].

#### Jalapin

##### Component organic acids and hydroxyl fatty acid

The crude jalapin fraction was subjected to alkaline hydrolysis to furnish organic acid and glycosidic acid fractions. The former fraction was methylated with diazomethane-ether and then examined by GC–MS, exhibiting two peaks identical to those of methyl *n*-decanoate and *n*-dodecanoate. The latter fraction furnished an aglycone fraction and a monosaccharide fraction upon acidic hydrolysis. The aglycone fraction was methylated with diazomethane-ether to furnish methyl ester (**1**) ([α]_D_ + 0.9°) of jalapinolic acid (**2**) [17].

##### Determination of the absolute configurations of hydroxyl fatty acids including jalapinolic acid

**Fig. 1:**
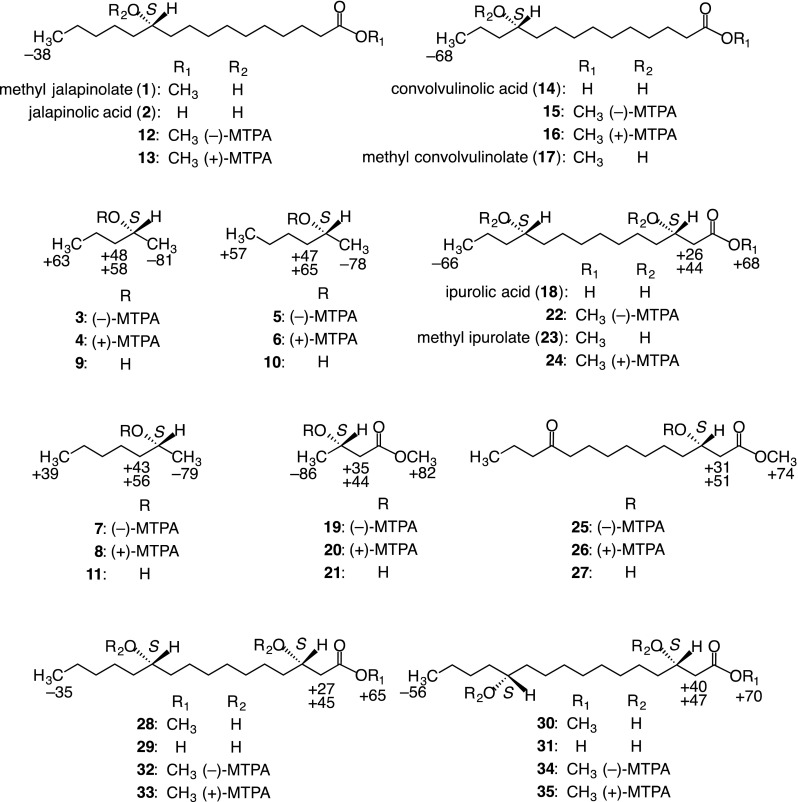
Structures of **1**–**35**, and values of ^1^H-NMR chemical shift difference (ppm) [∆*δ*: *δ*(−)-MTPA − *δ*(+)-MTPA ester (×10^−3^)] for MTPA esters (in CDCl_3_, **3**–**8**, **12**, **13**, **15**, **16**, **19**, **20**, **22**, **24**–**26**, **32**, **33**: 400 MHz; **34**, **35**: 600 MHz). Data were taken and reproduced from references Ono et al. [20, 23]

Mosher’s method [18] was used to determine the absolute configuration of **1**. The ^1^H-NMR spectrum of (+)-2-methoxy-2-trifluoromethylphenylacetate (MTPA ester) of the racemate of **1** as well as the ^1^H-NMR spectra of the (−)- and (+)-MTPA esters (**3**, **4**; **5**, **6**; **7**, **8**) of commercial 2*S*-pentanol (**9**), 2*S*-hexanol (**10**) and 2*S*-heptanol (**11**) as model compounds, indicated that Mosher’s method is applicable for the determination of the configuration of **1** (Fig. 1). This determination was accomplished using the chemical shift of the terminal methyl group, which is separated by four methylene groups from the asymmetric carbon. The chemical shift difference (∆*δ*) [*δ*(−)-MTPA ester–*δ*(+)-MTPA ester] of the H_3_-16 signals in the ^1^H-NMR spectra of the (−)- and (+)-MTPA esters (**12**, **13**) of **1** indicated the configuration at C-11 of **1** to be *S* [19, 20]. At the same time, Shibuya et al. determined the configuration of (+)-jalapinolic acid, which was obtained from the tuber of *Merremia mammosa*, to be *S* by syntheses of *S*- and *R*-jalapinolic acid via Sharpless asymmetric epoxidation [21].

In the same manner as **1**, the absolute configuration at C-11 of convolvulinolic acid (**14**), the aglycone of quamoclinic acid A, was determined to be *S* using the ∆*δ* value of (−)- and (+)-MTPA esters (**15**, **16**) of methyl convolvulinolate (**17**) ([α]_D_ + 1.0°) [20].

Ipurolic acid (**18**), the common aglycone of pharbitic acids C and D, has hydroxyl groups at C-3 as well as at C-11. The ^1^H-NMR spectral data of the (−)- and (+)-MTPA esters (**19**, **20**) of commercial methyl 3*S*-hydroxybutyrate (**21**), used as model compounds, indicated that the configuration at C-3 could be determined by the ∆*δ* values of the signals originating from the 2-methylene protons and ester methyl protons. Comparison of the ^1^H-NMR spectrum of (−)-MTPA ester (**22**) of methyl ipurolate (**23**) ([α]_D_ + 1.2°) with that of (+)-MTPA ester (**24**) suggested that the configurations at C-3 and C-11 of **18** are both *S*. The influence on their ∆*δ* values by interactions between two MTPA residues at C-3 and C-11 in **22** and **24** was ruled out as follows. Methyl 11-hydroxytetradec-2-enoate, which was prepared from crude pharbitic acid according to Okabe et al. [22], was hydrogenated to furnish **17**. In addition, the ^1^H-NMR spectra of (−)- and (+)-MTPA esters (**25**, **26**) of methyl 3-hydroxy-11-oxo-tetradecanoate (**27**), which was obtained by the partial oxidation of **23**, showed similar ∆*δ* values to those of **22** and **24** for signals due to 2-methylene protons and ester methyl protons. Thus, the configurations at C-3 and C-11 of **18** were both determined to be *S* [20].

The methyl ester (**28**) ([α]_D_ + 0.2°) of 3,11-dihydroxyhexadecanoic acid (**29**), the aglycone of pharbitic acid B, the methyl ester (**30**) of 3,12-dihydroxyhexadecanoic acid (**31**), and the aglycone of operculinic acid H, were converted into (−)- and (+)-MTPA esters (**32**, **33**; **34**, **35**). The ∆*δ* values for signals due to H_3_-16, Ha-2, Hb-2, and ester methyl protons were analogous to those of **22** and **24**. Therefore, the absolute configurations of **29** and **31** were concluded to be 3*S*, 11*S*, and 3*S*, 12*S*, respectively [20, 23]. The absolute configuration of **31** was compatible with that clarified by the enantioselective syntheses performed by Jakob and Gerlach [24].

Mosher’s method was thus found to be useful for the determination of the absolute configuration of a minute amount of hydroxyl fatty acids.

##### Component glycosidic acids

**Fig. 2:**
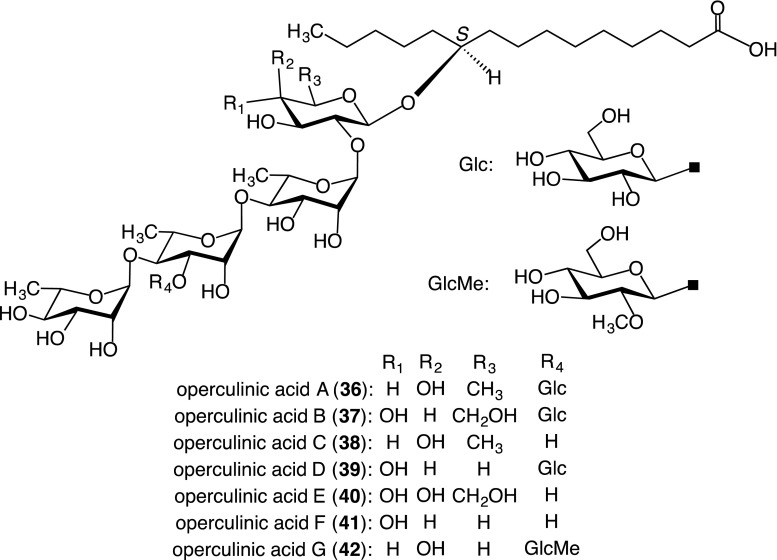
Structures of component glycosidic acids (**36**–**42**) of the crude jalapin fraction prepared from the root of *I. operculata*

The glycosidic acid fraction in MeOH was treated with diazomethane-ether. The concentrated reaction mixture was subjected to silica gel column chromatography and preparative HPLC on octadecyl silica (ODS), silica gel, and octyl silica columns to yield seven methyl esters of glycosidic acids, named operculinic acids A–G (**36**–**42**) [17, 19, 25]. Their structures were determined by NMR spectroscopy, negative-ion FAB-MS, and GC analyses of trimethylsilyl ethers of the thiazolidine derivatives [26] of monosaccharides provided on their acidic hydrolyses (Fig. 2). Among them, operculinic acid G (**42**) might be an artifact produced from operculinic acid A (**36**) during treatment with diazomethane [27].

##### Isolation of resin glycosides

The crude jalapin fraction was subjected to silica gel and ODS column chromatography and HPLC on silica, ODS, and octyl silica columns to yield operculins I–XVIII (**43**–**60**) [19, 28–30]. Preparative recycling HPLC was a useful method for the isolation of two constitutional isomers, in which the *n*-decanoyl group and *n*-dodecanoyl group were mutually interchanged between the same positions (**49** and **50**; **51** and **52**) [28, 29].

##### Structures of resin glycosides

**Fig. 3:**
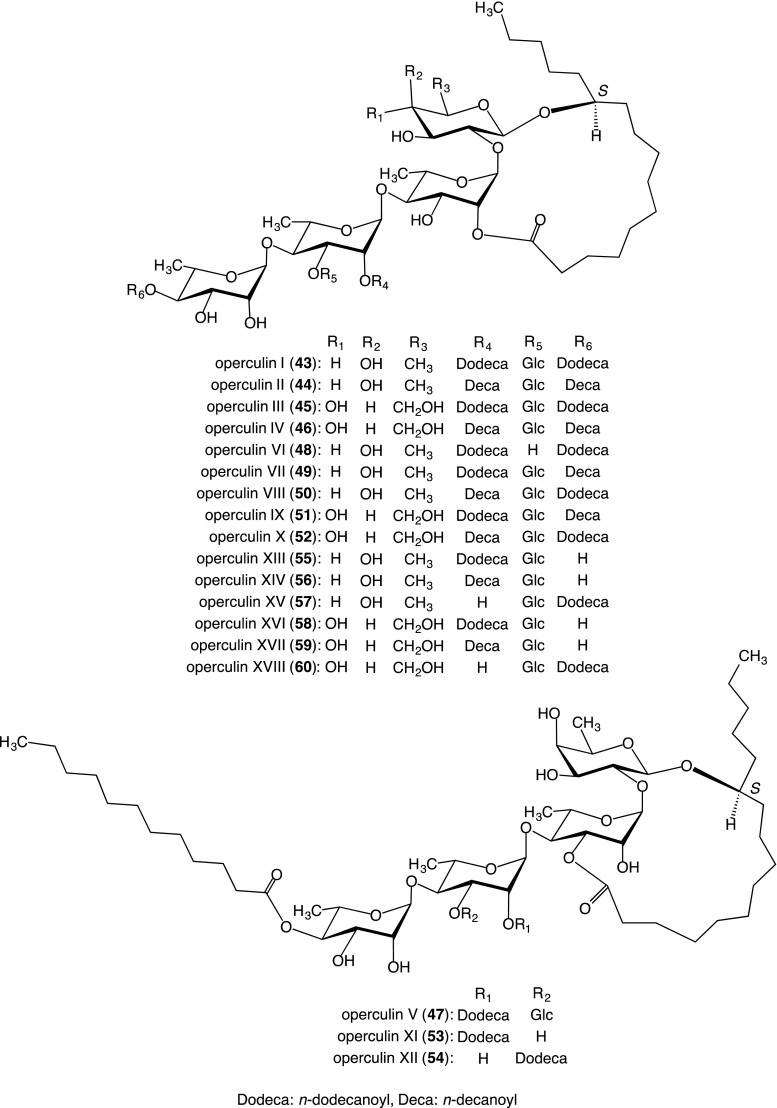
Structures of resin glycosides (**43**–**60**) isolated from the crude jalapin fraction prepared from the root of *I. operculata*

Operculin I (**43**) is composed of 2 mol of *n*-dodecanoic acid and 1 mol of **36** and is intramolecularly linked with a hydroxyl group of the sugar moiety to form a macrolactone structure, which was elucidated by using ^1^H- and ^13^C-NMR spectra, negative-ion FAB-MS, as well as analyses of the hydrolysates produced by alkaline hydrolysis. The site of each ester linkage in jalapinolic and *n*-dodecanoic acids were fixed by analyses of the acylation shifts observed in the ^1^H-NMR spectrum of **43** as well as peak assignments in the EI-MS of the peracetate of **43** and in the negative ion FAB-MS of **43**. From these data, the structure of **43** was defined as 11*S*-jalapinolic acid 11-*O*-β-d-glucopyranosyl-(1 → 3)-*O*-[4-*O*-*n*-dodecanoyl-α-l-rhamnopyranosyl-(1 → 4)]-*O*-(2-*O*-*n*-dodecanoyl)-α-l-rhamnopyranosyl-(1 → 4)-*O*-α-l-rhamnopyranosyl-(1 → 2)-β-d-fucopyranoside, intramolecular 1, 2″-ester, as shown in Fig. 3. [19, 28].

The structures of operculins II (**44**)–XVIIII (**60**) were determined using similar methods to those used for **43** (Fig. 3).

Compounds **43**–**60** are the first known resin glycosides to feature fatty acids, *n*-dodecanoic and/or *n*-decanoic acid, as component organic acids.

#### Convolvulin

##### Component organic acids

**Fig. 4:**
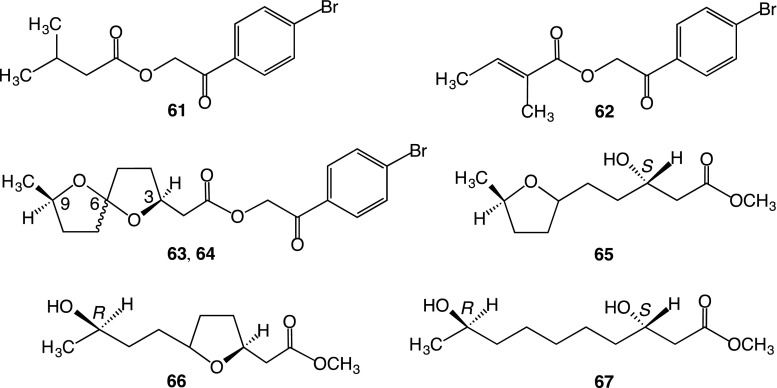
Structures of *p*-bromophenacyl esters (**61**–**64**) of component organic acids of the crude convolvulin fraction prepared from the root of *I. operculata* and hydrogenated products (**65**–**67**) of methyl exogonate

Alkaline hydrolysis of the crude convolvulin fraction afforded organic acid and glycosidic acid fractions. The organic acid fraction was esterified with *p*-bromophenacyl bromide, and the product was subjected to chromatographic separation to afford *p*-bromophenacyl isovalerate (**61**), *p*-bromophenacyl tiglate (**62**), and two compounds **63** and **64** (Fig. 4). Both **63** and **64** were identified as the *p*-bromophenacyl esters of the exogonic acid reported by Graf and Dahlke [14] using NMR and FD-MS data. Furthermore, it was elucidated that **63** and **64** are isomers and that interconversion between them occurred because of inversion at the C-6 spiro-center. The absolute configurations at C-3 and C-9 of exogonic acid were identified as *S* and *R*, respectively, by applying Mosher’s method to methyl 3-hydroxy-6,9-epoxydecanoate (**65**), methyl 9-hydroxy-3,6-epoxydecanoate (**66**), and methyl 3,9-dihydroxydecanoate (**67**), which were provided by hydrogenation of methyl exogonate. It was also found that this acid existed as a mixture of epimers that only differ in the spirocyclic carbon configuration at C-6 [23]. These results were consistent with those of the enantioselective syntheses performed by Lawson et al. [31].

##### Component glycosidic acids

The glycosidic acid fraction was treated with diazomethane-ether and subsequently subjected to chromatography to isolate the methyl ester (**68**), the alkaline hydrolysis of which afforded a glycosidic acid, named operculinic acid H (**69**). Acidic hydrolysis of **68** afforded an aglycone and a mixture of monosaccharides, which were composed of d-glucose and l-rhamnose. Methylation of the aglycone with diazomethane-ether yielded a methyl 3*S*,12*S*-dihydroxyhexadecanoate (**30**) [23].

**Fig. 5:**
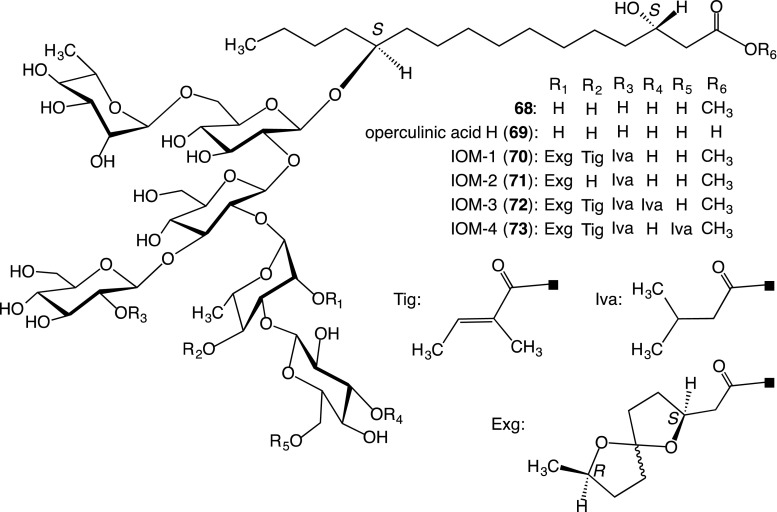
Structures of methyl ester (**68**) of operculinic acid H (**69**), **69** and acylated glycosidic acid methyl esters (**70**–**73**) generated from the crude convolvulin fraction from the root of *I. operculata* after treatment with indium(III) chloride in MeOH

The structure of operculinic acid H (**69**) was characterized as shown in Fig. 5 using the NMR data of **68** and its peracetate, negative-ion FAB-MS of **69**, HR-EI-MS of the permethylate of **68**, and structural analyses of the five hydrolysates produced by partial methanolysis of **68** [23]. Despite the large difference between the structures of the sugar moieties in **68** and Wagner’s operculinic acid [16], both compounds were considered to be identical. The organic acid and glycosidic acid components of the convolvulin fraction were different from those of the jalapin fraction obtained from the crude drug.

##### Structures of acylated glycosidic acid methyl esters

Despite numerous attempts, the isolation of pure resin glycosides from the crude convolvulin fraction has been unsuccessful. In consideration of previous results, it was evident that the component resin glycosides of the crude convolvulin fraction possessed at least one carboxyl group. Hence, the fraction was treated with indium(III) chloride in MeOH, which was reported to be a catalyst for mild methyl esterification of carboxylic acids by Mineno and Kansui [32]. The treated fraction exhibited a number of separate spots by TLC on silica gel and was successively separated by Sephadex LH-20 and silica gel column chromatography and HPLC using an ODS column, affording four compounds, referred to as IOM-1 (**70**)−IOM-4 (**73**) [33].

The ^1^H-NMR spectra of **70**–**73** showed that they consisted of mixtures of epimers that differed in the spirocyclic carbon configuration only at C-6 of the exogonoyl residue (Exg). Since HPLC using a naphthylethyl group-bonded silica (π-nap) column of **70**–**73** indicated that interconversion of the epimers of Exg in **70**–**73** was facile, structural analyses were carried out using a mixture of the epimers.

The structures of **70**–**73** were determined as shown in Fig. 5 using their NMR spectra, including HMBC and ^1^H-^1^H TOCSY spectra, negative-ion FAB-MS, and the structural analyses of their hydrolysates produced by alkaline hydrolysis and partial deacylation [33].

Compounds **70**–**73** were all regarded as the methyl ester monomers of the acylated glycosidic acid. Furthermore, negative-ion FAB-MS and HR-negative-ion ESI-TOF–MS of the crude convolvulin fraction exhibited intense ion peaks at *m/z* 1659 and 1659.8010 (calculated for C_77_H_127_O_38_^−^, 1659.8011), corresponding to the values of an [M − H]^−^ ion peak of the demethylated derivatives of **72** and **73**, respectively. In addition, no distinct peak was detected in the region of *m/z* 1700–4000 in the negative-ion FAB-MS of the crude convolvulin fraction. Therefore, a part of the crude convolvulin fraction might be a mixture of monomers composed of free carboxylic acid forms corresponding to **72** and **73** [33].

Thus, we consider that methyl esterification of carboxylic acids via indium(III) chloride catalysis is a useful tool for structural investigations involving convolvulins.

### *Pharbitis nil*

Pharbitis Semen, the seed of *Pharbitis nil* Choisy, is used as a crude purgative drug, and its resin glycoside is a typical Mayer’s convolvulin. Asahina et al. reported that alkaline hydrolysis of the crude glycoside named pharbitin gave (+)-2-methylbutyric acid, tiglic acid, nilic acid, and a glycosidic acid named pharbitic acid, which was composed of ipurolic acid, d-glucose, and l-rhamnose [34–36]. In 1970, Okabe et al. isolated two glycosidic acids, named pharbitic acids C (**74**) and D (**75**), along with valeric, tiglic, nilic, and (+)-2-methylbutyric acids, as components of the alkaline hydrolysis products of pharbitin [22, 37–39].

#### Component organic acids

Alkaline hydrolysis of pharbitin furnished organic and glycosidic acid fractions. The GC of the organic acid fraction revealed the presence of 2-methylbutyric, tiglic, and nilic acid in a molar ratio of approximately 17:1:11. Furthermore, the organic acid fraction was acylated with *p*-bromophenacyl bromide followed by chromatographic separation to yield *p*-bromophenacyl 2*S*-methylbutyrate and *p*-bromophenacyl nilate, which was found to be a mixture of the 2*R*, 3*R*- and the 2*S*, 3*S*-forms with a ratio of approximately 6:5 based on the ratio of H_3_-5 signal intensities obtained from the ^1^H-NMR spectrum of its (−)-MTPA ester [40, 41].

##### Component hydroxyl fatty acids and monosaccharides

Acidic hydrolysis of the glycosidic acid fraction yielded hydroxyl fatty acid and monosaccharide fractions. Methylation of the hydroxyl fatty acid fraction yielded methyl 3*S*,11*S*-ipurolate (**23**) and methyl 3*S*,11*S*-dihydroxyhexadecanoate (**28**). The component monosaccharides were determined to be l-rhamnose, d-quinovose, and d-glucose [40].

##### Component glycosidic acids

**Fig. 6:**
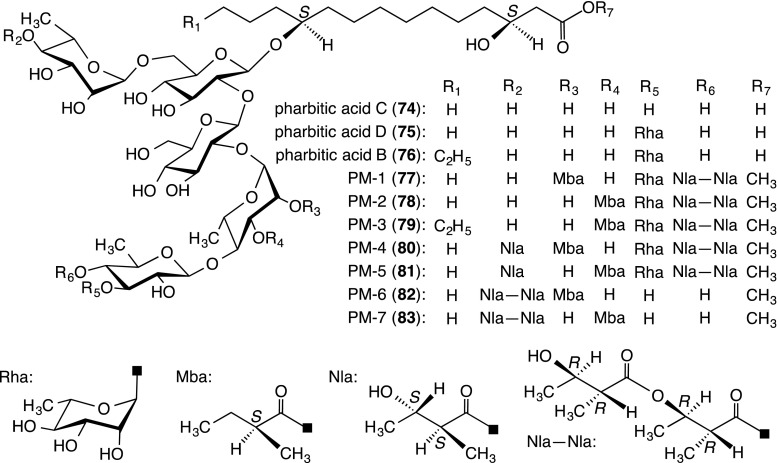
Structures of component glycosidic acids (**74**–**76**) of pharbitin and acylated glycosidic acid methyl esters (**77**–**83**) generated from pharbitin by treatment with indium(III) chloride in MeOH

The glycosidic acid fraction was converted into *p*-phenylphenacyl ester, and chromatography yielded three glycosidic acid esters. The alkaline hydrolysis of each ester furnished a new glycosidic acid, named pharbitic acid B (**76**), along with pharbitic acids C (**74**) and D (**75**), whose structures were corrected by NMR and negative-ion FAB-MS data as well as the EI-MS data for the permethylates of **74** and **75** [40] (Fig. 6). Using NMR and negative-ion FAB-MS, **76** was concluded to be a homolog of **75**, in which 3*S*,11*S*-ipuroric acid (**18**) of the aglycone was replaced by 3*S*,11*S*-dihydroxyhexadecanoic acid (**29**) [40].

##### Structures of acylated glycosidic acid methyl esters

Pharbitin was treated with indium(III) chloride in MeOH, in a similar manner to the treatment of the crude convolvulin fraction from *I. operculata*. This treated pharbitin was successively subjected to Diaion HP20, Sephadex LH-20, and silica gel column chromatography and HPLC using an ODS column to afford seven compounds, temporarily named PM-1 (**77**)−PM-7 (**83**) [42].

The structures of **77**–**83** were determined as indicated in Fig. 6 using similar methods as those used for **70**–**73** [33]. Compounds **77**–**83** were all monomers of methyl esters of glycosidic acids esterified with several organic acids at the sugar moiety. In addition, negative-ion FAB-MS of pharbitin exhibited ion peaks at *m/z* 1551, 1451, and 1305, which corresponded to the values of [M − H]^−^ ion peaks of the demethylated derivatives of **80**, **78**, and **82**, respectively. However, for pharbitin, no intense ion peaks were observed in the *m/z* 1580–3500 region. Therefore, a part of the pharbitin was considered to be a mixture of monomers composed of free carboxylic acid forms corresponding to **77**–**83**. Furthermore, it should be noted that **80** and **81** had 2*R*,3*R*-nilic acid and its enantiomer as the component organic acids in each molecule [42].

### *Quamoclit pennata*

*Quamoclit pennata* is native to tropical regions of South America, and is cultivated primarily as an ornamental plant. This seed contains both jalapins and convolvulins.

#### Jalapin

##### Component organic acids and glycosidic acid

**Fig. 7:**
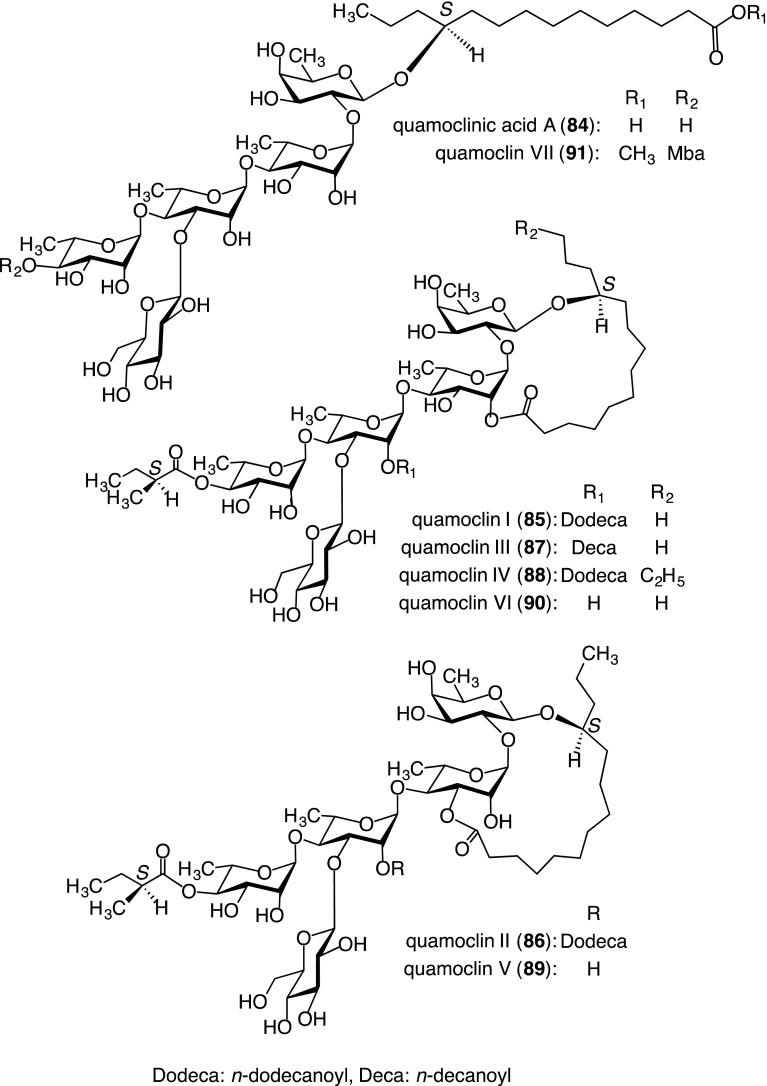
Structures of component glycosidic acid (**84**) and resin glycosides (**85**–**91**) isolated from the crude jalapin fraction prepared from the seeds of *Q. pennata*

The component organic acids were elucidated to be 2*S*-methylbutyric, *n*-decanoic, and *n*-dodecanoic acids. On the other hand, the glycosidic acid fraction yielded a new glycosidic acid, named quamoclinic acid A (**84**), whose structure was determined to be 11*S*-convolvulinolic acid 11-*O*-β-d-glucopyranosyl-(1 → 3)-*O*-[α-l-rhamnopyranosyl-(1 → 4)]-*O*-α-l-rhamnopyranosyl-(1 → 4)-*O*-α-l-rhamnopyranosyl-(1 → 2)-β-d-fucopyranoside on the basis of spectroscopic data and chemical evidence (Fig. 7). It is noteworthy that the aglycone (11*S*-convolvulinolic acid) of **84** differs from jalapinolic acid [43].

##### Structures of resin glycosides

Six resin glycosides with macrolactone structures, named quamoclins I (**85**)–VI (**90**) and one acylated glycosidic acid methyl ester, named quamoclin VII (**91**), were isolated. Their structures were assigned using similar methods as those employed for operculins. Compounds **85**–**90** were the first examples of jalapins with an organic acid (2*S*-methylbutyric acid) and fatty acid components (*n*-decanoic or *n*-dodecanoic acids) [43, 44] (Fig. 7). Compound **91** was considered to be an artifact produced from **89** and/or **90** during the extraction and isolation procedures.

#### Convolvulin

##### Component organic acids, hydroxyl fatty acids, and monosaccharides

The alkaline hydrolysis products of the crude convolvulin fraction were fractionated into organic acid and glycosidic acid fractions. The former fraction was composed of isobutyric, 2*S*-methylbutyric, tiglic, 2*R*,3*R*-nilic, 7*S*-hydroxydecanoic, and 7*S*-hydroxydodecanoic acids.

Acidic hydrolysis of the glycosidic acid fraction gave aglycone and monosaccharide fractions. Methylation of the former with diazomethane-ether yielded methyl 7*S*-hydroxydecanoate and methyl 3*S*,11*S*-ipurolate (**23**). The monosaccharide fraction was composed of d-glucose, d-fucose, d-quinovose, and l-rhamnose [41].

##### Structures of glycosidic acids

**Fig. 8:**
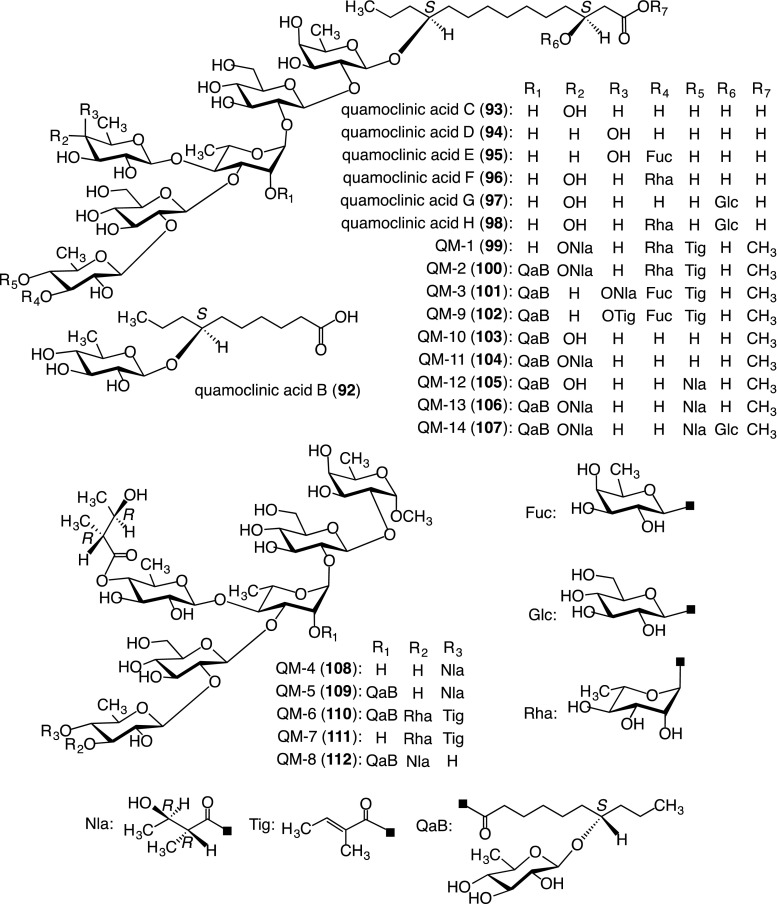
Structures of component glycosidic acids (**92**–**98**) of the crude convolvulin fraction prepared from the seeds of *Q. pennata* and acylated glycosidic acid methyl esters (**99**–**107**) and acylated methyl glycosides (**108**–**112**) generated from the crude convolvulin fraction by treatment with indium(III) chloride in MeOH

The glycosidic acid fraction furnished five new glycosidic acids, named quamoclinic acids B (**92**)–H (**98**). These compounds were characterized on the basis of spectroscopic data as well as chemical evidence (Fig. 8). Compounds **95**–**97** were the first examples of heptaglycosides of glycosidic acid, and **98** was the first octaglycoside of glycosidic acid. Furthermore, **97** and **98** were the first known glycosidic acids to feature bisdesmosides with sugar linkages at the C-3 position of ipurolic acid as well as at its C-11 position [41, 45].

Except for 2*S*-methylbutyric acid, the component organic and glycosidic acids were different from those obtained from the crude jalapin fraction of this seed.

##### Acylated glycosidic acid methyl esters and acylated methyl glycosides

Treatment of the crude convolvulin fraction with indium(III) chloride in MeOH afforded nine acylated glycosidic acid methyl esters, named QM-1 (**99**), QM-2 (**100**), QM-3 (**101**), QM-9 (**102**), QM-10 (**103**), QM-11 (**104**), QM-12 (**105**), QM-13 (**106**), and QM-14 (**107**), and five acylated methyl glycosides, named QM-4 (**108**), QM-5 (**109**), QM-6 (**110**), QM-7 (**111**), and QM-8 (**112**). Their structures were elucidated on the basis of spectroscopic data and chemical conversions [46–48] (Fig. 8).

Since two acylated trisaccharides closely related to the resin glycosides were previously reported as natural constituents of the seeds of *Cuscuta chinensis* (Convolvulaceae) [49], **108**–**112** are presumably formed from the corresponding acylated saccharides with a reducing end during treatment with indium(III) chloride in MeOH. It was therefore presumed that one of the reasons for the difficulty in isolating convolvulin from the crude convolvulin fraction of *Q. pennata* was the co-existence of the acylated saccharides with a reducing end, because the α- and β-anomers easily reach equilibrium.

### *Calystegia soldanella*

*Calystegia soldanella* Roem. et Schult. (Convolvulaceae) is distributed widely on the sandy beaches of seas and lakes in temperate regions of the world. The roots of this plant are used for the treatment of arthritis. Gasper reported the isolation and structural elucidation of two resin glycosides, soldanellines A and B, as chemical constituents of the root [50, 51].

#### Component organic acids and glycosidic acids

**Fig. 9:**
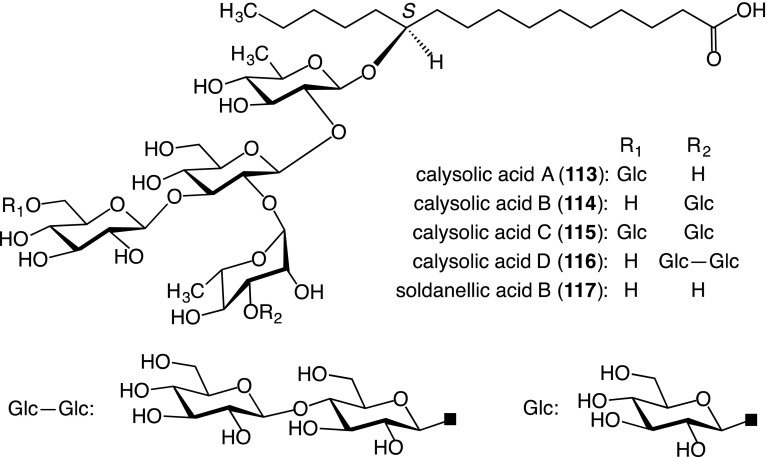
Structures of component glycosidic acids (**113**–**117**) of the crude resin glycoside fraction prepared from the leaves, stems, and roots *of C. soldanella*

Alkaline hydrolysis of the crude resin glycoside fraction obtained from the MeOH extract of the leaves, stems, and roots gave four new glycosidic acids, named calysolic acids A (**113**)–D (**116**), along with one known glycosidic acid, soldanellic acid B (**117**) [51], and three organic acids, 2*S*-methylbutyric, tiglic, and 2*S*,3*S*-nilic acids. The structures of the new glycosidic acids were characterized on the basis of spectroscopic data and chemical evidence [52] (Fig. 9).

##### Structures of resin glycosides

**Fig. 10:**
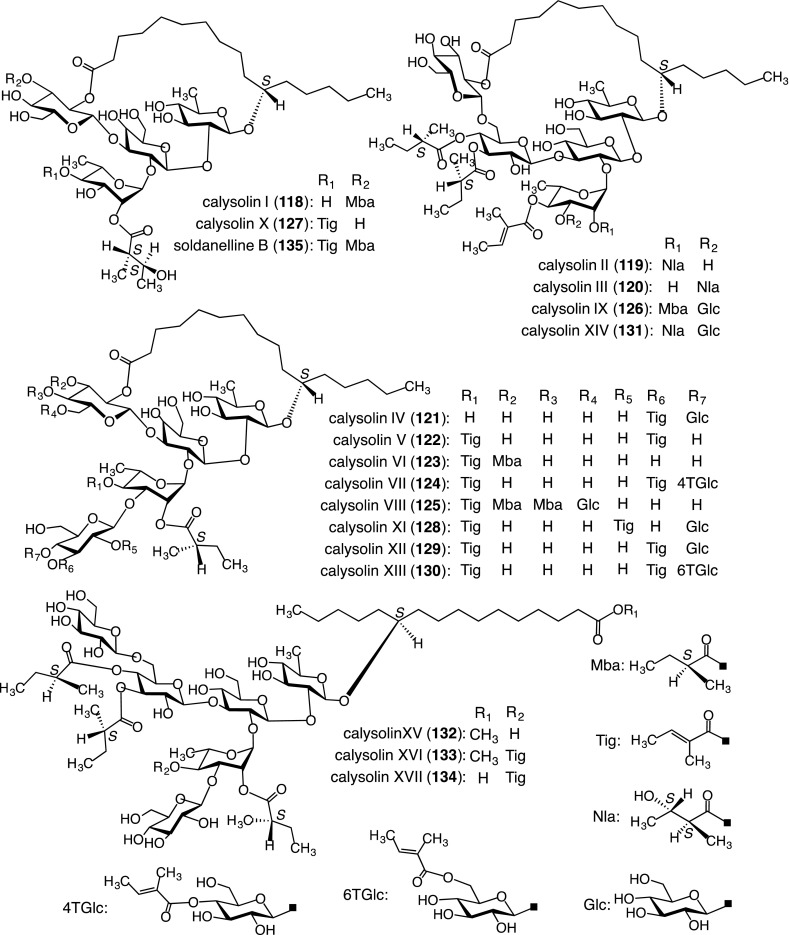
Structures of resin glycosides (**118**–**135**) isolated from the leaves, stems, and roots *of C. soldanella*

Seventeen new resin glycosides, named calysolins I–XVII (**118**–**134**), were isolated from the crude resin glycoside fraction along with soldanelline B (**135**). Their structures were determined on the basis of spectroscopic data and chemical evidence [53–56] (Fig. 10). They consisted of two types, one possessing macrolactone structures (**118**–**131**, **135**) and the other type with non-macrolactone structures (**132**–**134**). Compounds **132** and **133** were methyl esters of acylated glycosidic acids, and **134** was the free carboxylic acid form of **133**. Furthermore, macrolactone structures fall into two types, one having a 22-membered ring (**118**, **121**–**125**, **128**–**130**, **135**) and the other with a 27-membered ring (**119**, **120**, **126**, **131**). It is possible that **132**–**134** are artifacts formed from the macrolactone resin glycosides during the extraction and/or isolation procedures. Compounds **121**, **124**–**126**, and **128**–**131** were the first examples of hexaglycosides of resin glycosides with a macrolactone structure. Additionally, **118**–**135** showed antiviral activity toward herpes simplex type 1 [54–56].

### *I. muricata*

The seeds of *I. muricata* (L.) Jacq. are used as a laxative and carminative in the folk medicine of India. In previous papers, Noda et al. reported that alkaline hydrolysis of the crude resin glycoside fraction of the seeds of *I. muricata* furnished three organic acids, isobutyric, 2*S*-methylbutyric, and 2*R*,3*R*-nilic acid, along with a glycosidic acid fraction composed of l-rhamnose, d-fucose, d-quinovose, and 11*S*-jalapinolic acid (**2**). They also discussed the isolation and structural elucidation of three glycosidic acids including muricatic acids A–C [20, 57, 58]. Furthermore, eight genuine resin glycosides, muricatins I–VIII, which possessed characteristic macrolactone structures, were reported [58, 59].

#### Structure of resin glycoside

**Fig. 11:**
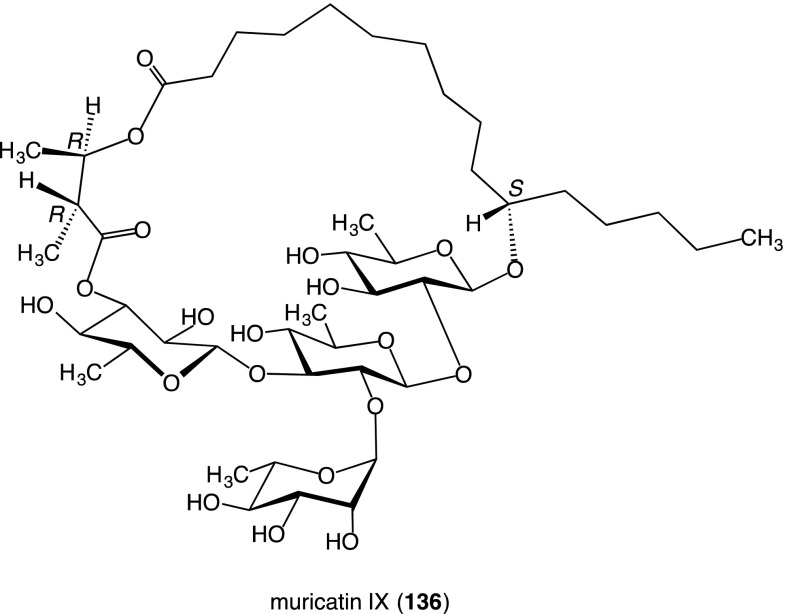
Structure of muricatin IX (**136**) isolated from the seeds of *Ipomoea muricata*

A new resin glycoside, named muricatin IX (**136**), was isolated from the seeds of *I. muricata*. The structure of **136** was determined on the basis of spectroscopic data as well as chemical evidence. It should be noted that **136** is different from all the resin glycosides isolated so far, in that the carboxyl group of its aglycone moiety is linked with a hydroxyl group of the organic acid, which was attached to a sugar moiety by ester linkage, to form a macrocyclic ester ring [60] (Fig. 11).

## Conclusions

Resin glycosides were classified into two groups by Mayer in 1855, according to their solubility in ether. These groups consisted of jalapins, which are ether-soluble, and convolvulins, which are ether-insoluble. This classification has since been conventionally used. Almost all ether-soluble resin glycosides hitherto isolated had common intermolecular macrocyclic ester structures composed of 1 mol of a variety of acylated glycosidic acids, of which some examples were ester-type dimers. On the other hand, the crude ether-insoluble resin glycosides fraction of the root of *I. operculata*, pharbitin, and the crude ether-insoluble resin glycosides fraction from seeds of *Q. pennata*, which were typical Mayer’s convolvulins, show tailing or broad peaks on TLC and HPLC, suggesting that they differ from ether-soluble resin glycosides in chemical structure, and their crude ether-insoluble resin glycoside fractions furnished acylated glycosidic acid methyl ester monomers after treatments with indium(III) chloride in MeOH. In addition, the *m/z* values of intense ion peaks in the negative-ion FAB-MS of the crude ether-insoluble resin glycoside fractions obtained from the root of *I. operculata* and pharbitin corresponded to the values of [M − H]^−^ ion peaks of demethylated derivatives of the some of their acylated glycosidic acid methyl esters. However, no intense ion peaks were observed in their dimer region. Therefore, a part of the crude ether-insoluble resin glycoside fraction might be a mixture of acylated glycosidic acid monomers with free carboxylic acid forms. On the other hand, calysolins IV, XI, and XIV, which have a macrolactone structure, hardly dissolve in ether. In addition, muricatin IX is different from all the resin glycosides isolated so far, in that the carboxyl group of its aglycone moiety linked with a hydroxyl group of the organic acid, which is attached to a sugar moiety by an ester linkage, to form a macrocyclic ester ring. Furthermore, the root of *I. operculata* and the seeds of *Q. pennata* contain both ether-soluble resin glycosides and ether-insoluble resin glycosides. The component organic and glycosidic acids of ether-soluble resin glycoside fractions of these materials were different from those of the corresponding ether-insoluble resin glycoside fraction, respectively. Therefore, classification of resin glycosides into two groups is considered to be meaningful.

As mentioned above, many findings concerning the structures of resin glycosides have been obtained so far. Taking those findings into account, it would be preferable that the terms ‘jalapin’ and ‘convolvulin’ should not be used based on their solubility in ether as classified by Mayer, but rather to describe structural groups, i.e., the resin glycosides having intramolecular cyclic structures and the acylated glycosidic acid monomers and polymers with free carboxylic acid forms, respectively [61].

## References

[1] Shellard EJ (1961). The chemistry of some Convolvulaceous resins part 1. Vera cruz jalap. Plant Med.

[2] Mayer W (1855). Ueber die sogenannten jalappaharze. Justus Liebigs Ann Chem.

[3] Mannich C, Schumann P (1938). Jalap resin and its principal constituent, convolvulin. Arch Pharm.

[4] Noda N, Ono M, Miyahara K, Kawasaki T (1987). Resin glycosides. I. Isolation and structure elucidation of orizabin-I, II, III and IV, genuine resin glycosides from the root of *Ipomoea orizabensis*. Tetrahedron.

[5] Noda N, Tsuji K, Kawasaki T, Miyahara K, Hanazono H, Yang C-R (1995). A novel resin glycoside, meremin (tuguajalapin × dimer), from *Merremia hungaiensis*. Chem Pharm Bull.

[6] Bah M, Pereda-Miranda R (1997). Isolation and structural characterization of new glycolipid ester-type dimers from the resin of *Ipomoea tricolor* (Convolvulaceae). Tetrahedron.

[7] Escalante-Sánchez E, Pereda-Miranda R (2007). Batatins I and II, ester-type dimers of acylated pentasaccarides from the resin glycosides of sweet potato. J Nat Prod.

[8] Castañeda-Gómez J, Pereda-Miranda R (2011). Resin glycosides from the herbal drug jalap (*Ipomoea purga*). J Nat Prod.

[9] Rosas-Rameírez D, Escalante-Sánchez E, Pereda-Miranda R (2011). Batatins III–VI, glycolipid ester-type dimers from *Ipomoea batatas*. Phytochemistry.

[10] Castañeda-Gómez J, Figueroa-González G, Jacobo N, Pereda-Miranda R (2013). Purgin II, a resin glycoside ester-type dimer and inhibitor of multidrug efflux pumps from *Ipomoea purga*. J Nat Prod.

[11] Rosas-Raíez D, Pereda-Miranda R (2013). Resin glycosides from the yellow-skinned variety of sweet potato (*Ipomoea batatas*). J Agri Food Chem.

[12] Rosas-Raíez D, Pereda-Miranda R (2015). Batatins VIII–XI, glycolipid ester-type dimers from *Ipomoea batatas*. J Nat Prod.

[13] Corona-Castañeda B, Rosas-Ramírez D, Castañeda-Gómez J, Aparico-Cuevas MA, Fragoso-González G, Pereda-Miranda R (2016). Resin glycosides from *Ipomoea wolcottiana* as modulators of the multidrug resistance phenotype in vitro. Phytochemistry.

[14] Graf E, Dahlke E (1964). Structure elucidation of exogonic acid. Chem Ber.

[15] Graf E, Dahlke E, Voigtlander HW (1965). Convolvulin; new fragment units and differentiation reactions. Arch Pharm Ber Dtsch Pharm Ges.

[16] Wagner H, Kazmaier P (1977). Structure of the operculinic acid from the resin of *Ipomoea operculata*. Phytochemistry.

[17] Ono M, Kawasaki T, Miyahara K (1989). Resin glycosides. V. Identification and characterization of the component organic and glycosidic acids of the ether-soluble crude resin glycosides (“jalapin”) from Rhizoma Jalapae Braziliensis (roots of *Ipomoea operculata*). Chem Pharm Bull.

[18] Dale JA, Mosher HS (1973). Nuclear magnetic resonance enantiomer regents. Configurational correlations via nuclear magnetic resonance chemical shifts of diastereomeric mandelate, *O*-methylmanndelate, and α-methoxy-α-trifluoromethylphenylacetate (MTPA) esters. J Am Chem Soc.

[19] Ono M, Kubo K, Miyahara K, Kawasaki T (1989). Operculin I and II, new ether-soluble resin glycosides (“jalapin”) with fatty acid ester groups from Rhizoma Jalapae Braziliensis (roots of *Ipomoea operculata*). Chem Pharm Bull.

[20] Ono M, Yamada F, Noda N, Kawasaki T, Miyahara K (1993). Resin glycosides. XVIII. Determination by Mosher’s method of the absolute configurations of mono- and dihydroxyfatty acids originated from resin glycosides. Chem Pharm Bull.

[21] Shibuya H, Kawashima K, Baek NI, Narita N, Yoshikawa M, Kitagawa I (1989). Synthesis of (11*S*)-(+)- and (11*R*)-(−)-jalapinolic acids. A revision of chemical structure of merremosides B and D. Chem Pharm Bull.

[22] Okabe H, Koshito N, Tanaka K, Kawasaki T (1971). Studies on resin glycosides. II. Unhomogeneity of “pharbitin” and isolation and partial structures of pharbitic acids C and D, the major constituents of “pharbitic acid”. Chem Pharm Bull.

[23] Ono M, Nishioka H, Fukushima T, Kunimatu H, Mine A, Kubo H, Miyahara K (2009). Components of ether-insoluble resin glycoside (rhamonvolvulin) from Rhizoma Jalapae Braziliensis. Chem Pharm Bull.

[24] Jakob B, Gerlach H (1996). Relative and absolute configuration of 3,12-dihydroxypalmitic acids. Liebings Ann.

[25] Ono M, Fukunaga T, Kawasaki T, Miyahara K (1990). Resin glycosides. VIII. Four new glycosidic acids, operculinic acids D, E, F and G, of the ether-soluble crude resin glycosides (“jalapin”) from Rhizoma Jalapae Braziliensis (roots of *Ipomoea operculata*). Chem Pharm Bull.

[26] Hara S, Okabe H, Mihashi K (1987). Gas-liquid chromatographic separation of aldose enantiomers as trimethylsilyl ethers of methyl 2-(polyhydroxyalkyl)-thiazolidine-4(*R*)-carboxylates. Chem Pharm Bull.

[27] Aritomi M, Kawasaki T (1970). Partial methylation with diazomethane of the sugar moiety of some *C*- and *O*-d-glucopyranosides. Chem Pharm Bull.

[28] Ono M, Nishi M, Kawasaki T, Miyahara K (1990). Resin glycosides. IX. Operculins I, II, V, VII and VIII, new ether-soluble resin glycosides of Rhizoma Jalapae Braziliensis (roots of *Ipomoea operculata*). Chem Pharm Bull.

[29] Ono M, Kawasaki T, Miyahara K (1991). Resin glycosides. XI. Operculins III, IV, IX, X, XVI, XVII and XVIII, new ether-soluble resin glycosides of Rhizoma Jalapae Braziliensis (the root of *Ipomoea operculata*). Chem Pharm Bull.

[30] Ono M, Fujimoto K, Kawata M, Fukunaga T, Kawasaki T, Miyahara K (1992). Resin glycosides. XIII. Operculins VI, XI, XII, XIII, XIV and XV, the ether-soluble resin glycosides (jalapin) from Rhizoma Jalapae Braziliensis (roots of *Ipomoea operculata*). Chem Pharm Bull.

[31] Lawson EN, Jamie JF, Kitching W (1992). Absolute stereochemistry of exogonic acid. J Org Chem.

[32] Mineno T, Kansui H (2006). High yielding methyl esterification catalyzed by indium(III) chloride. Chem Pharm Bull.

[33] Ono M, Oda S, Yasuda S, Mineno T, Okawa M, Kinjo J, Miyashita H, Yoshimitsu H, Nohara T, Miyahara K (2017). Acylated glycosidic acid methyl esters generated from the convolvulin fraction of Rhizoma Jalapae Braziliensis by treatment with indium(III) chloride in methanol. Chem Pharm Bull.

[34] Asahina Y, Terada S (1919). Constituents of the seeds of *Pharbitis nil* chois. Yakugaku Zasshi.

[35] Asahina Y, Shimizu T (1922). Constituents of the seeds of *Pharbitis nil* chois. II. Yakugaku Zassh.

[36] Asahina Y, Nakanishi Y (1925). Constituents of the seeds of *Pharbitis nil* chois. III. Yakugaku Zasshi.

[37] Okabe H, Kawasaki T (1970). Structures of pharbitic acids C and D. Tetrahedron Lett.

[38] Kawasaki T, Okabe H, Nakatsuka I (1971). Studies on resin glycosides. I. Reinvestigation of the components of pharbitin, a resin glycoside of the seeds of *Pharbitis nil* CHOISY. Chem Pharm Bull.

[39] Okabe H, Kawasaki T (1972). Studies on resin glycosides. III. Complete structures of phabitic acids C and D. Chem Pharm Bull.

[40] Ono M, Noda N, Kawasaki T, Miyahara K (1990). Resin glycosides. VII. Reinvestigation of the component organic and glycosidic acids of pharbitin, the crude ether-insoluble resin glycoside (“convolvulin”) of Pharbitidis Semen (seeds of *Pharbitis nil*). Chem Pharm Bul.

[41] Ono M, Takagi-Taki Y, Honda-Yamada F, Noda N, Miyahara K (2010). Components of ether-insoluble resin glycoside (convolvulin) from seeds of *Quamoclit pennata*. Chem Pharm Bull.

[42] Ono M, Takigawa A, Mineno T, Yoshimitsu H, Nohara T, Ikeda T, Fukuda-Teramachi E, Noda N, Miyahara K (2010). Acylated glycosides of hydroxy fatty acid methyl esters generated from the crude resin glycoside (pharbitin) of seeds of *Pharbitis nil* by treatment with indium(III) chloride in methanol. J Nat Prod.

[43] Ono M, Kuwabata K, Kawasaki T, Miyahara K (1992). Resin glycosides. XIV. Quamoclins I–IV, new ether-soluble resin glycosides (“jalapin”) from the seeds of *Quamoclit pennata*. Chem Pharm Bull.

[44] Ono M, Takaki Y, Takatsuji M, Akiyama K, Okawa M, Kinjo J, Miyashita H, Yoshimitsu H, Nohara T (2012). Three new resin glycosides and a new tetrahydropyran derivative from the seeds of *Quamoclit pennata*. Chem Pharm Bull.

[45] Ono M, Imao M, Miyahara K (2010). Two new glycosidic acids, quamoclinic acids G and H, of the resin glycosides (convolvulin) from the seeds of *Quamoclit pennata*. Chem Pharm Bull.

[46] Akiyama K, Mineno T, Okawa M, Kinjo J, Miyashita H, Yoshimitsu H, Nohara T, Ono M (2013). Three acylated glycosidic acid methyl esters and two acylated methyl glycosides generated from the convolvulin fraction of seeds of *Quamoclit pennata* by treatment with indium(III) chloride in methanol. Chem Pharm Bull.

[47] Akiyama K, Yamamoto K, Mineno T, Okawa M, Kinjo J, Yoshimitsu H, Nohara T, Ono M (2014). Five new resin glycoside derivatives isolated from the convolvulin fraction of seeds of *Quamoclit pennata* after treatment with indium(III) chloride in methanol. Chem Pharm Bull.

[48] Ono M, Akiyama K, Yamamoto K, Mineno T, Okawa M, Kinjo J, Miyashita H, Yoshimitsu H, Nohara T (2014). Four new acylated glycosidic acid methyl esters isolated from the convolvulin fraction of seeds of *Quamoclit pennata* after treatment with indium(III) chloride in methanol. Chem Pharm Bull.

[49] Miyahara K, Du X-M, Watanabe M, Sugiura C, Yahara S, Nohara T (1996). Resin glycosides. XXIII. Two novel acylated trisaccharides related to resin glycoside from the seeds of *Cuscuta chiensis*. Chem Pharm Bull.

[50] Gasper EM (1999). New pentasaccharide macrolactone from the European convolvulaceae *Calysegia soldanella*. Tetrahedron Lett.

[51] Gasper EM (2001). Soldanelline B: the first acylated nonlinear tetrasaccharide macrolactone from the Europeane Convolvulaceae plant *Calystegia soldanella*. Eur J Org Chem.

[52] Takigawa A, Setoguchi H, Okawa M, Kinjo J, Miyashita H, Yokomizo K, Yoshimitsu H, Nohara T, Ono M (2011). Identification and characterization of component organic and glycosidic acids of crude resin glycoside fraction from *Calystegia soldanella*. Chem Pharm Bull.

[53] Takigawa A, Muto H, Kabata K, Okawa M, Kinjo J, Yoshimitsu H, Nohara T, Ono M (2011). Calysolins I—IV, resin glycosides from *Calystegia soldanella*. J Nat Prod.

[54] Ono M, Takigawa A, Kanemaru Y, Kawakami G, Kabata K, Okawa M, Kinjo J, Yokomizo K, Yoshimitsu H, Nohara T (2014). Calysolins V–IX, resin glycosides from *Calystegia soldanella* and their antiviral activity toward herpes. Chem Pharm Bull.

[55] Ono M, Kawakami G, Takigawa A, Kabata K, Okawa M, Kinjo J, Yokomizo K, Yoshimitsu H, Nohara T (2014). Calysolins X–XIII, resin glycosides from *Calystegia soldanella* and their antiviral activity toward herpes simplex virus. Chem Pharm Bull.

[56] Ono M, Takigawa A, Muto H, Kabata K, Okawa M, Kinjo J, Yokomizo K, Yoshimitsu H, Nohara T (2015). Antiviral activity of four new resin glycosides calysolins XIV–XVII from *Calystegia soldanella* against herpes simplex virus. Chem Pharm Bull.

[57] Noda N, Kobayashi H, Miyahara K, Kawasaki T (1988). Resin glycosides. II. Identification and characterization of the component organic and glycosidic acids of the crude resin glycoside from the seeds of *Ipomoea muricata*. Chem Pharm Bull.

[58] Noda N, Nishi M, Miyahara K, Kawasaki T (1988). Resin glycosides. IV. Two new resin glycosides, muricatins VII and VIII, from the seeds of *Ipomoea muricata*. Chem Pharm Bull.

[59] Noda N, Kobayashi H, Miyahara K, Kawasaki T (1988). Resin glycosides. III. Isolation and structural study of the genuine resin glycosides, muricatins I–VI, from the seeds of *Ipomoea muricata*. Chem Pharm Bull.

[60] Ono M, Taketomi S, Kakiki Y, Yauda S, Okawa M, Kinjo J, Yoshimitsu H, Nohara T (2016). A new resin glycoside, muricatin IX, from the seeds of *Ipomoea muricata*. Chem Pharm Bull.

[61] Ono M, Nakagawa K, Kawasaki T, Miyahara K (1993). Resin glycosides. XIX. Woodrosins I and II, ether-insoluble resin glycosides from the stems of *Ipomoea tuberosa*. Chem Pharm Bull.

